# Successful Tampon Pulpotomy in a Molar With an Endodontic Lesion: A Case Report

**DOI:** 10.7759/cureus.55006

**Published:** 2024-02-26

**Authors:** Saeed Asgary

**Affiliations:** 1 Iranian Center for Endodontic Research, Research Institute of Dental Sciences, Shahid Beheshti University of Medical Sciences, Tehran, IRN

**Keywords:** calcium-enriched mixture cement, vital pulp therapy, apical periodontitis, irreversible pulpitis, tampon pulpotomy

## Abstract

Vital pulp therapy (VPT) has emerged as an alternative approach to root canal treatment (RCT) for managing cases with irreversible pulpitis/apical periodontitis, aiming to preserve pulp vitality and promote healing and regeneration of pulpal tissues. The tampon approach, which entails the placement of endodontic biomaterials over the pulpal wound to mechanically tamponade uncontrollable bleeding, shows promise as a technique within VPT. A 32-year-old female patient presented with severe/lingering pain in the lower left quadrant. Clinical/radiographic examinations confirmed symptomatic irreversible pulpitis and symptomatic apical periodontitis in the first right lower molar; radiographic examination exhibited an endodontic lesion for the mesial root and periodontal ligament (PDL) widening for the distal root. The patient opted for VPT; however, despite several attempts to achieve hemostasis using various solutions, including NaOCl, hemorrhage persisted. Therefore, a layer of freshly mixed calcium-enriched mixture cement was applied using a dry cotton pellet, resulting in bleeding control. Then, a permanent restoration was placed. Follow-up examinations revealed the resolution of symptoms and the one-year radiographic examination showed complete healing of the endodontic lesion. The successful outcomes highlight the effectiveness of tampon pulpotomy in managing irreversible pulpitis and associated apical lesions. Tampon pulpotomy offers several advantages, including preserving healthy pulp tissue, reduced invasiveness, and immediate hemorrhage control. This technique presents an alternative to more invasive procedures, such as RCT, and promotes patient satisfaction through a simplified treatment approach. Further clinical trials are needed to validate the findings of this case report and establish the long-term success rates of tampon pulpotomy.

## Introduction

Irreversible pulpitis, characterized by inflammation of the dental pulp leading to severe and persistent tooth pain, is a common endodontic condition. While root canal treatment (RCT) has conventionally been the mainstay for managing irreversible pulpitis, minimally invasive vital pulp therapies (VPTs) have emerged as promising alternatives aimed at preserving pulp vitality and facilitating the healing and regeneration of dental pulp tissues [1]. This paradigm shift underscores the necessity for innovative approaches, particularly in cases where traditional treatments may prove inadequate.

In cases of irreversible pulpitis, the inflammatory process can extend beyond the roots, resulting in the development of apical periodontitis/lesion, characterized by inflammation and tissue destruction in the periapical region, often accompanied by radiographic evidence of an endodontic lesion [2]. Effective and minimally invasive strategies are crucial to address both pulpal and periapical aspects of the endodontic disease process.

Among emerging VPT techniques, the tampon approach has shown promise, particularly in cases of prolonged or excessive bleeding (PB) following dental pulp amputation in primary and permanent teeth [3-5]. This innovative procedure involves the application of an endodontic biomaterial, such as calcium-enriched mixture (CEM; BioniqueDent, Tehran, Iran) cement, directly over the pulpal wound to mechanically control bleeding and establish an optimal environment for pulpal healing. Recent research, including a study on a molar with irreversible pulpitis and a prior VPT failure, has demonstrated the long-term success of tampon pulpotomy [5]. The tampon approach exerts direct physical pressure on the dental pulpal wound, triggering physiological clotting mechanisms and promoting the formation of an intravascular fibrin clot at the cutting surface, thus facilitating VPT procedures and initiating the healing cascade.

While various biomaterials have gained prominence in contemporary endodontics, calcium-enriched mixture (CEM) cement has emerged as a standout candidate, garnering significant attention for its versatile applications in VPTs [6,7]. Its unique composition, which includes calcium oxide, sulfur trioxide, phosphorous pentoxide, and silicon dioxide, distinguishes it from mineral trioxide aggregate and other traditional materials, giving it several desirable properties, such as excellent sealing ability, antimicrobial activity, and the capacity to stimulate dentinogenesis and cementogenesis [8,9]. Moreover, CEM cement demonstrates low cytotoxicity and high alkalinity, facilitating the induction of reparative dentin formation and thereby enhancing the long-term success of VPT procedures. These favorable characteristics position CEM cement as a promising alternative to conventional endodontic materials, offering clinicians a reliable option for preserving pulp vitality and fostering tissue regeneration.

In this case report, we present a clinical case of tampon pulpotomy in a tooth diagnosed with irreversible pulpitis and associated apical periodontitis/lesion. The objective of this report is to showcase the efficacy of tampon pulpotomy in managing endodontic pathologies and underscore its potential as a valuable technique in VPT.

## Case presentation

**Figure 1:**
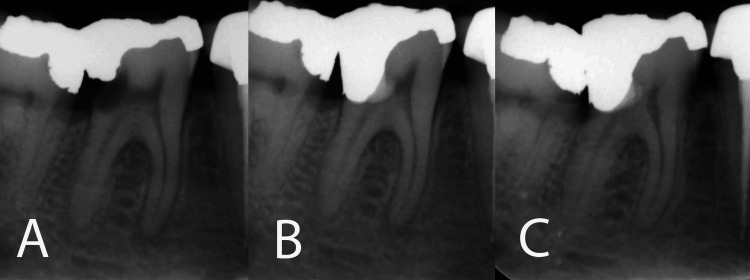
Radiographic progression of the treated case (A) Preoperative radiograph showing the involved tooth with deep caries cavity and a radiolucent apical lesion/PDL widening in the mesial/distal roots, respectively. (B) Immediate postoperative radiograph demonstrating the placement of calcium-enriched mixture cement after tampon pulpotomy. (C) One-year follow-up radiograph revealing the reestablishment of a normal PDL, indicating successful pulpal healing and resolution of apical pathology. Notably, a thick dentinal bridge is evident in front of the CEM biomaterial, separating the related pulp chamber of the mesial roots.

A 32-year-old healthy female presented to the Mehr Dental Clinic with the chief complaint of severe/lingering pain in the lower left quadrant spontaneously or upon eating/drinking icy liquids. Clinical examination revealed the presence of previous amalgam restorations in all premolars and molars in the lower left quadrant, along with a recurrent carious lesion in the first molar. The symptomatic first molar exhibited tenderness to percussion and an extended response lasting more than 20 seconds to cold spray compared to other healthy mature teeth. Periodontal probing indicated normal findings. Radiographic evaluation showed an amalgam-restored tooth with a deep caries cavity, a radiolucent apical lesion in the mesial root, and periodontal ligament (PDL) widening in the distal root (Figure 1A). The patient had no significant medical history. Based on the clinical and radiographic assessments, the tooth was diagnosed with symptomatic irreversible pulpitis associated with symptomatic apical periodontitis. The treatment options, including root canal treatment or VPT, were discussed with the patient, who preferred VPT and provided informed consent.

Under local anesthesia with 2% lidocaine containing 1:80,000 epinephrine (Darupakhsh, Tehran, Iran) and rubber dam isolation, the previous restoration of tooth #36 and the caries lesion were completely excavated using a high-speed sterile round diamond bur (Tizkavan, Tehran, Iran) with water cooling. This procedure extensively exposed the pulp, i.e., more than 2 mm, and the distal root canal orifice was identified. However, the mesial part of the pulp chamber, protected by intact dentin, remained undamaged, and the coronal dental pulp over the mesial canals was left unmanipulated. Despite attempts to achieve hemostasis using a cotton pellet soaked in chlorhexidine (Iran Najo Pharmaceutical Co., Tehran, Iran) for ~5 minutes and repeated episodes with 5.25% sodium hypochlorite (NaOCl; Pars Co., Tehran, Iran) for ~2 minutes, excessive hemorrhage persisted. In the absence of hemostasis, the pulpal tissue was tamponed by applying a ~2 mm layer of freshly mixed CEM cement (BioniqueDent, Tehran, Iran) with a creamy consistency, effectively stopping the bleeding. Immediately upon application of the CEM cement and using a dry cotton pellet, hemorrhage ceased due to the mechanical occlusion of the pulp wound. Subsequently, an amalgam restoration (Cinalux Faghihi, Tehran, Iran) was placed with meticulous caution. Finally, a postoperative periapical radiograph was taken to evaluate the quality of the VPT procedures (Figure 1B).

The treated tooth was monitored during follow-up visits. Within a day, the patient reported the disappearance of painful symptoms. At regular three-month recall appointments, the tooth was functional and asymptomatic; the response to the cold test was within the normal range. Radiographic examination after a year revealed healing of the apical lesion and the reestablishment of a normal periodontal ligament (Figure 1C).

## Discussion

The management of irreversible pulpitis and associated apical periodontitis presents a clinical challenge, with traditional approaches like RCT being commonly employed. However, VPT has gained attention as an alternative technique aiming to preserve pulp vitality and promote tissue healing/regeneration [10]. In this case report, the successful application of tampon pulpotomy in a tooth with irreversible pulpitis and apical periodontitis/lesion was demonstrated, highlighting its effectiveness in managing these conditions. The resolution of the patient’s symptoms and radiographic evidence of complete healing in the periapical region underscore the success of this technique [11]. Furthermore, the absence of pain and restoration of a normal periodontal ligament at the one-year recall validate the efficacy of tampon pulpotomy in symptom relief and promoting healing.

In this specific case, the decision to utilize the tampon approach stemmed from encountering persistent hemorrhage during the pulpotomy procedure, despite efforts to achieve hemostasis using conventional methods. The immediate hemostasis achieved by tamponizing freshly mixed CEM cement with a dry cotton pellet enabled uninterrupted treatment [4,5], highlighting the efficacy of the tampon approach in managing cases with prolonged or excessive hemorrhage. This contributes to the expanding literature on VPT and deepens our comprehension of its applications.

When examining the implications of the tampon approach hypothesis, it is critical to delve into the terminology used in VPT modalities, particularly the differentiation between conventional methods and the innovative tampon approach. Additionally, it is vital to emphasize the significance of addressing the delayed clotting process in cases of irreversible pulpitis with PB. Rather than following the traditional sequence of wasting time, bleeding control, and achieving hemostasis of the pulpal wound before covering the pulp stump with a suitable biomaterial, the concept of the tampon approach introduces a promising strategy for managing such challenging scenarios efficiently. This tailored approach aims to achieve hemostasis mechanically and immediately while promoting pulp healing/regeneration, without unnecessary delay in clinical procedures. Notably, a recent cohort study has shown that the tampon approach yields successful outcomes comparable to cases with normal hemostasis during VPTs, further supporting its efficacy and feasibility in routine clinical practice [12].

Tampon pulpotomy offers several advantages over RCT: It preserves healthy pulp tissue, essential for long-term tooth function; it diminishes the necessity for invasive procedures like pulpectomy or extraction, particularly beneficial in primary teeth [4], thereby conserving natural tooth structure and minimizing patient discomfort; and it provides a time-saving alternative, allowing for an immediate VPT procedure and restoration placement, thereby enhancing patient satisfaction.

While this case report offers valuable insights into tampon pulpotomy, its generalizability is limited. The necessity for additional research, including larger case series and randomized clinical trials, to establish broader applicability is recognized. Inherent limitations, such as sample size and the absence of a control group, are intrinsic to case reports. Future studies should build upon these initial observations to advance VPT.

## Conclusions

This case report highlights the effectiveness of tampon pulpotomy in managing irreversible pulpitis and associated apical periodontitis/lesion. By preserving the healthy pulp tissue and promoting pulpal/periapical healing, tampon pulpotomy offers a viable treatment option with advantages such as immediate hemorrhage control, preservation of tooth vitality, and reduced invasiveness. Further research and clinical studies are warranted to validate these findings and establish the long-term success rates of tampon pulpotomy.
